# Artificial Intelligence Methods in the Detection of Oral Diseases on Pantomographic Images—A Systematic Narrative Review

**DOI:** 10.3390/jcm14093262

**Published:** 2025-05-07

**Authors:** Katarzyna Zaborowicz, Maciej Zaborowicz, Katarzyna Cieślińska, Agata Daktera-Micker, Marcel Firlej, Barbara Biedziak

**Affiliations:** 1 Department of Orthodontics and Facial Malformations, Poznan University of Medical Sciences, Bukowska 70, 60-812 Poznań, Poland; kcieslinska@ump.edu.pl (K.C.); agatamicker@ump.edu.pl (A.D.-M.); marcel-firlej@wp.pl (M.F.); biedziak@ump.edu.pl (B.B.); 2 Department of Biosystems Engineering, Poznan University of Life Sciences, Wojska Polskiego 50, 60-627 Poznań, Poland

**Keywords:** artificial intelligence in dentistry, digital pantomographic imaging, caries detection, periapical lesions, cysts and tumours diagnosis, neural networks in medical imaging, diagnostic accuracy of AI models

## Abstract

**Background:** Artificial intelligence (AI) is playing an increasingly important role in everyday dental practice and diagnosis, especially in the area of analysing digital pantomographic images. Through the use of innovative and modern algorithms, clinicians can more quickly and accurately identify pathological changes contained in digital pantomographic images, such as caries, periapical lesions, cysts, and tumours. It should be noted that pantomographic images are one of the most commonly used imaging modalities in dentistry, and their digital analysis enables the construction of AI models to support diagnosis. **Objectives:** This paper presents a systematic narrative review of studies included in scientific articles from 2020 to 2025, focusing on three main diagnostic areas: detection of caries, periapical lesions, and cysts and tumours. The results show that neural network models, such as U-Net, Swin Transformer, and CNN, are most commonly used in caries diagnosis and have achieved high performance in lesion identification. In the case of periapical lesions, AI models such as U-Net and Decision Tree also showed high performance, surpassing the performance of young dentists in assessing radiographs in some cases. **Results:** The studies cited in this review show that the diagnosis of cysts and tumours, on the other hand, relies on more advanced models such as YOLO v8, DCNN, and EfficientDet, which in many cases achieved more than 95% accuracy in the detection of this pathology. The cited studies were conducted at various universities and institutions around the world, and the databases (case databases) analysed in this work ranged from tens to thousands of images. **Conclusions:** The main conclusion of the literature analysis is that, thanks to its accessibility, speed, and accuracy, AI can significantly assist the work of physicians by reducing the time needed to analyse images. However, despite the promising results, AI should only be considered as an enabling tool and not as a replacement for the knowledge of doctors and their long experience. There is still a need for further improvement of algorithms and further training of the network, especially in identifying more complex clinical cases.

## 1. Introduction

With the advancement of information technology and computer image analysis, more and more doctors are utilising artificial intelligence in the diagnostic process of patients. Commonly performed digital X-ray images in dentistry allow for the creation of extensive databases that can be used to develop artificial neural network models. The developed algorithms assist doctors in analysing X-ray images and detecting pathological changes such as dental caries, periapical lesions, cysts, and tumours.

Although panoramic radiographs are not as precise as CBCT (Cone Beam Computed Tomography), they enable the identification of hard tissues in the oral cavity. Despite certain limitations in imaging specific oral structures, panoramic radiography remains a highly popular and widely accessible diagnostic method. The use of digital techniques has significantly reduced radiation exposure, making it one of the most commonly performed examinations in dentistry.

The widespread use of this method, its ease of execution, and the minimal radiation exposure for patients have contributed to panoramic radiographs being frequently used in computer image analysis for developing neural network models. These models assist doctors in fast and effective diagnostics, facilitating the detection of abnormalities in the analysed patient images.

The aim of this systematic narrative review of the current state of knowledge is to present the latest trends in dentistry, where artificial intelligence methods are increasingly being employed for the analysis of panoramic radiographs.

## 2. Materials and Methods

The paper was prepared in accordance with The PRISMA 2020 statement guidelines found at https://www.prisma-statement.org/, accessed on 22 March 2025, (see Supplementary Materials). This study analysed the most frequently cited articles in the field of dentistry and artificial intelligence, published in the Scopus database between 2020 and 2025. In this review, we have limited ourselves to analysing the Scopus database, due to its popularity and wide access. The citation criterion is one of the filters in this database, widely used in ongoing analyses of the popularity of scientific papers. It should be noted that the popularity criterion is biased and does not indicate the high scientific quality of the work. The Scopus database was selected due to its high-quality indexing. The research was conducted in February 2025. The articles were categorised into three groups. The first category was defined by the keywords dental caries, panoramic radiograph, and artificial intelligence [1,2,3,4,5,6,7,8,9,10]. The second category included periapical lesions, panoramic radiograph, and artificial intelligence [1,6,9,11,12,13,14,15,16,17]. The third and final category was based on the keywords: tumours and cysts, panoramic radiograph, and artificial intelligence [18,19,20,21,22,23,24,25].

For each of the three categories, the most frequently cited studies were selected, excluding literature reviews, short reports, and book chapters. Articles written in languages other than English were excluded from the analysis. The selected articles are publicly accessible.

In the first category, 15 articles were retrieved, out of which the 10 most frequently cited ones were selected (Table 1). In the second category, there were 16 articles, and again, the 10 most frequently cited papers were chosen (Table 2). In the third category, only 8 articles were found, two of which had no citations. Therefore, it was decided to include these two articles in the review as well (Table 3).

The analysed data have been compiled into tables (Table 4, Table 5, Table 6, Table 7, Table 8, Table 9, Table 10, Table 11 and Table 12). For each category, three tables have been developed. The first table contains information regarding the research objective, the number of panoramic radiographs used, the distribution by gender, and the location where this study was conducted (Table 4, Table 7 and Table 10). The second table includes details on the type of neural network, test quality, and error rate, as well as the results and conclusions drawn from the presented data (Table 5, Table 8 and Table 11). The third table presents the risk of bias assessment using PROBAST (Table 6, Table 9 and Table 12).

Analysing the available scientific studies on the detection of dental caries in digital panoramic images using artificial intelligence (AI) methods, significant differences can be observed in the number of analysed images, the models used, and the diagnostic effectiveness of these models.

In terms of the number of digital panoramic images used, the study with the smallest dataset was conducted by the team of Zadrożny et al. [1]. This study included only 30 images, which is a relatively small sample size, especially when compared to other cited works. In contrast, the largest dataset was used by Gardiyanoğlu et al. [5], who analysed 8138 panoramic images. The number of images used in other cited studies ranged between these values, from 30 to 8138—for instance, Zhu et al. [2] based their study on 1996 images, while Asci et al. [10] utilised 6075 panoramic images.

Regarding the most commonly used AI models, different types of models were employed. However, various variants of the U-Net neural network dominated, including its advanced versions such as Nested U-Net, U-Net++, and U-Net3+. Some studies also used other techniques and models, such as PyRadiomics ver. 1 combined with a classical artificial neural network (ANN) [3], which aimed to perform computer-based extraction of advanced radiomic features. On the other hand, the study by Zadrożny et al. [1] used Diagnocat, a commercial AI tool for panoramic image analysis. In the study by Zhou et al. [8], the Swin Transformer model was applied and further optimised for detecting dental caries in children.

Analysing the diagnostic effectiveness of individual models, the best results were achieved by the PyRadiomics ver. 1 + ANN model for detecting radiation-induced caries, which had a very high AUC value of 0.9886, indicating an almost perfect ability to distinguish between healthy and diseased teeth. Similarly, high effectiveness was demonstrated by U-Net3+, achieving 95% accuracy in the study by Alharbi et al. [7]. The Swin Transformer, tested by Zhou et al. [8], reached an AUC of 0.9223, also indicating high model performance.

Conversely, the lowest diagnostic parameters were observed for Diagnocat in the study by Zadrożny et al. [1], which obtained a low intraclass correlation coefficient (ICC = 0.681) for caries detection, meaning its results were not fully reliable. Additionally, Zhu et al. [2] noted that their AI model had the lowest effectiveness, specifically in caries detection, with an AUC value of only 0.772.

Regarding the division of studied individuals by gender, most analyses did not consider this variable as a factor affecting caries detection effectiveness. An exception was the study by Faria et al. [3], which included 15 patients, of whom 13 were men and 2 were women, indicating a gender imbalance in the dataset. In other studies, such as Asci et al. [10], particularly those focused on paediatric diagnostics, different stages of dental development were analysed, but without a clear gender division.

In conclusion, the analysis of the cited studies suggests that the effectiveness of caries detection in panoramic images using AI largely depends on the applied model. The best results were achieved by advanced models such as PyRadiomics + ANN and U-Net3+, whereas simple CNNs and Diagnocat had significantly lower effectiveness. Additionally, the number of analysed images (training cases) varied significantly between studies, which could have influenced the obtained results. Most studies did not analyse differences in caries diagnostics between genders, which may represent a research gap and provide a basis for further analyses and quality assessments of AI models.

Research on the detection of periapical lesions in panoramic radiographs using artificial intelligence (AI) has varied in terms of dataset sizes, applied models, and detection effectiveness. Some of the cited studies also considered the division of patients by gender.

Regarding the number of analysed images, the largest dataset was presented and used in the study by Endres et al. [11], where a total of 2902 digital panoramic radiographs were examined. This large sample size allowed for a precise determination of the prevalence of periapical lesions and an assessment of associated risk factors. Conversely, the smallest number of digital panoramic images was used in the study by Zadrożny et al. [1], where only 30 panoramic radiographs were evaluated, which may have limited the representativeness of the results.

Among the analysed studies, the most commonly used model was the U-Net neural network, which proved effective in segmenting periapical lesions. Notably, in the studies by Bayrakdar et al. [12] and Song et al. [13], the use of this architecture achieved high detection accuracy. In the study by Bayrakdar et al. [12], the U-Net model achieved a sensitivity of 0.92, a precision of 0.84, and an F1-score of 0.88, making it one of the best-performing models in this research area. Alternatively, in the studies by Kazimierczak et al. [14] and Zadrożny et al. [1], the Diagnocat AI system was used and evaluated for its diagnostic effectiveness compared to radiologists’ assessments.

In terms of model performance, the best results were obtained in the study by Bayrakdar et al. [12], where U-Net achieved the highest F1-score of 0.88, confirming its high precision in detecting periapical lesions. In contrast, the lowest performance was observed in the AI used in the study by Kazimierczak et al. [14], where the F1-score for panoramic radiographs was only 32.73%, indicating significant difficulties in the correct classification of lesions.

Some studies also included gender-based analysis. In the study by Herbst et al. [17], which analysed 1071 digital panoramic radiographs, it was found that men had periapical lesions more frequently (52.5%) compared to women (44.8%). Similar analyses were conducted in the study by Turosz et al. [9], although no specific differences in lesion prevalence between men and women were provided. Most other studies did not address this aspect.

In summary, among the analysed studies, the highest detection accuracy for periapical lesions was demonstrated by the U-Net model in the study by Bayrakdar et al. [12], while the lowest results were recorded in the study by Kazimierczak et al. [14]. The largest dataset was used in the study by Endres et al. [11], enabling a more precise assessment of lesion prevalence. Some studies, such as the one by Herbst et al. [17], also considered gender differences, showing a higher frequency of lesions in men. This analysis confirms that AI, particularly deep neural network-based models, has significant potential as a supportive tool in radiological diagnostics in dentistry.

In the analysed articles on the detection of cysts and tumours in digital panoramic radiographs using artificial intelligence, a significant diversity was observed in both the number of images used and the AI models applied. Each study in the reviewed papers approached the problem slightly differently, but certain common patterns can be identified, allowing conclusions to be drawn.

Regarding the number of images, the largest dataset was used in the study by Tajima et al. [22], where a Deep Convolutional Neural Network (DCNN) model was trained on as many as 7160 digital panoramic radiographs, with an additional 100 images used for testing the system. As a result, this model achieved a very high accuracy of 98.3% and a sensitivity of 94.4%, indicating its high effectiveness in detecting pathological changes. On the other hand, the smallest dataset was found in the study by Okazaki et al. [19], where only 150 images were used, which could have influenced the model’s training and its parameters. In this case, the accuracy was 70%, and the sensitivity was 70.8%.

The most commonly used model in the studies was YOLO (You Only Look Once), which appeared in three articles. In the study by Yang et al. [18], YOLO v2 was applied, achieving a precision of 0.707 and a sensitivity of 0.680, which was not the highest result compared to other approaches. However, in the study by Rašić et al. [21], the newer YOLO v8 model from 2023 was used, yielding significantly better results—the model’s precision reached 95.2%, and the mean Average Precision at IoU = 50% (mAP@50) was 97.5%, one of the highest scores in the analysed studies. This demonstrates how much newer and more advanced algorithm versions can improve diagnostic accuracy.

Apart from YOLO, several studies utilised DCNN and EfficientNet/EfficientDet. DCNN-based models proved to be effective—in the study by Tajima et al. [22], an accuracy of 98.3% was achieved, while in the study by Ha et al. [23], the EfficientDet model reached an effectiveness exceeding 99.8%, the highest result among all analysed studies. On the other end of the spectrum, AlexNet was used in the study by Okazaki et al. [19]—this model had the lowest effectiveness, with its precision and sensitivity not exceeding 70%, suggesting that older CNN architectures may struggle with the analysis of digital panoramic radiographs.

An interesting aspect was the presence of gender division in some studies. In the study by Yang et al. [18], demographic data were included, indicating that 32% of the training dataset comprised women, while 68% were men. Similarly, in the study by Okazaki et al. [19], it was reported that 51 patients were women and 99 were men. In the remaining reviewed studies, there was a lack of information on gender distribution, which could be an important factor in assessing the effectiveness of AI models in different patient groups.

The conclusions from this analysis indicate that the best results were achieved by DCNN and EfficientDet models, particularly when trained on large datasets. AlexNet proved to be the least effective, suggesting that older neural network architectures may not be suitable for such tasks. It was observed that larger datasets led to better model performance, and the lack of gender-based analysis in most studies may point to a potential gap in evaluating the impact of demographic characteristics on diagnostic accuracy.

In summary, the studies clearly show that the use of artificial intelligence for detecting cysts and tumours in panoramic radiographs has great potential, but the effectiveness of AI models depends on both the algorithm used and the amount of data utilised for training and testing. The best results are achieved by modern neural network architectures, such as EfficientDet and the latest YOLO versions, which may indicate the direction of future research in this field.

## 3. Summary

Artificial intelligence (AI) is playing an increasingly significant role in dental diagnostics, particularly in the analysis of digital panoramic radiographs. The use of advanced algorithms enables the detection of various conditions, including caries, periapical lesions, cysts, and tumours. Studies conducted at various universities worldwide have demonstrated AI’s high effectiveness in analysing radiological images.

The most commonly used AI models in research include convolutional neural networks (CNN), U-Net, Swin Transformer, and deep learning models. Results indicate that AI can achieve diagnostic accuracy comparable to or even higher than that of dentists. For instance, the Swin Transformer model achieved an AUC accuracy of 92.23% in caries detection. Although some studies report high diagnostic accuracy of AI models, these results should be interpreted with caution, as they strongly depend on the model architecture, quality and size of the training data, and evaluation methods.

In caries diagnostics, neural networks analysed thousands of images, achieving high sensitivity and precision values. The U-Net3+ model reached 95% accuracy, while the ANN model demonstrated an AUC of 0.9869 in predicting radiotherapy-related caries. These studies highlight the potential of AI in improving diagnostic processes.

The analysis of periapical lesions also demonstrated the high effectiveness of AI models. Research conducted at institutions such as Harvard University, Charité-Universitätsmedizin Berlin, and Seoul National University showed that AI could even outperform less experienced dentists in radiograph assessment. The AI Insights software achieved a classification accuracy of 99% and a detection efficiency of 95% for periapical lesions.

The most frequently used models in this category include U-Net, CNN, and Diagnocat AI software. The Decision Tree model (Shallow ML) achieved an F1-score of 0.90, confirming its high effectiveness in detecting periapical lesions. In some cases, AI outperformed experienced specialists, underscoring its growing importance in dental diagnostics.

Cyst and tumour diagnostics also benefit from AI, with deep learning models demonstrating high effectiveness. Studies were conducted at Yonsei University Dental Hospital, Hiroshima University Hospital, Clinical Hospital Dubrava, and other institutions. The YOLO v8 model achieved a precision of 95.2% and an mAP@50 of 97.5% in detecting lesions in the mandible.

Models such as DCNN, EfficientDet, and EfficientNet proved particularly effective. The DCNN model achieved an accuracy of 98.3% and an F-score of 0.966 in cyst detection. EfficientDet reached 99.8% accuracy in distinguishing pathological cysts from Stafne cysts, while EfficientNetB6 effectively classified mucous cysts (Table 13).

The study results indicate that AI has enormous potential in radiological diagnostics and can significantly assist doctors in identifying pathological changes in the oral cavity. The speed of analysis and the ability to process large datasets make AI a valuable tool in dentistry. AI can be especially beneficial in areas with limited access to specialists, enabling remote diagnostics and monitoring of patients’ oral health.

Despite AI’s high effectiveness, researchers emphasise that algorithms should be considered as support for doctors rather than a replacement. There is still a need for model optimisation, particularly in detecting more challenging cases such as periapical lesions. Despite the impressive results reported in the literature, several limitations must be acknowledged when considering the practical implementation of artificial intelligence in dental diagnostics. One of the primary challenges is the issue of overfitting—many models demonstrate high accuracy on training datasets, yet their ability to generalise to new, unseen clinical data remains uncertain. This concern is particularly relevant in studies that were conducted on relatively small datasets, which may not adequately represent the full spectrum of clinical variability.

Another critical limitation is the lack of clinical validation for most of the AI models presented. Many studies were performed in controlled laboratory settings, often without direct comparison to the decisions of experienced clinicians in real-world scenarios. The absence of standardised protocols for integrating AI systems into daily clinical workflows significantly hinders their broader adoption.

The “black-box” nature of many deep learning models also poses a substantial challenge. AI-generated diagnostic decisions are frequently not transparent or easily interpretable by clinicians, which may reduce trust in these tools and complicate the identification of system errors. As a result, there is a growing interest in explainable artificial intelligence (XAI), which aims to increase the transparency and interpretability of diagnostic models.

Finally, the ethical and societal implications of increasing reliance on automated diagnostic systems cannot be overlooked. Questions arise regarding accountability for diagnostic errors—whether it lies with software developers, end-users, or implementing institutions. Moreover, there is a concern that over-reliance on AI might reduce critical thinking skills in younger clinicians, who may become overly dependent on algorithmic outputs.

Therefore, further research is needed not only to improve and validate AI technologies but also to assess their impact on clinical decision-making, patient safety, and compliance with ethical and legal standards in healthcare.

## Figures and Tables

**Table 1:** The selected articles from the first category.

Number	First Author	Title	Journal	Year of Publication	Number of Citations
1.	Zadrożny Ł.	Artificial Intelligence Application in Assessment of Panoramic Radiographs	Diagnostics, 12(1), 224	2022	41
2.	Zhu J.	Artificial intelligence in the diagnosis of dental diseases on panoramic radiographs:a preliminary study	BMC Oral Health, 23(1), 358	2023	28
3.	De Araujo Faria V.	Prediction of Radiation-Related Dental Caries Through PyRadiomics Features and Artificial Neural Network on Panoramic Radiography	Journal of Digital Imaging, 34(5), pp. 1237–1248	2021	20
4.	Kabir T.	A comprehensive artificial intelligence framework for dental diagnosis and charting	BMC Oral Health, 22(1), 480	2022	18
5.	Gardiyanoğlu E.	Automatic Segmentation of Teeth, Crown–Bridge Restorations, Dental Implants, Restorative Fillings, Dental Caries, Residual Roots, and Root Canal Fillings on Orthopantomographs: Convenience and Pitfalls	Diagnostics, 13(8), 1487	2023	10
6.	Güneç H.G.	Comparison of artificial intelligence vs. junior dentists’ diagnostic performance based on caries and periapical infection detection on panoramic images	Quantitative Imaging in Medicine and Surgery, 13(11), pp. 7494–7503	2023	7
7.	Alharbi S.S.	Detection of Cavities from Dental Panoramic X-ray Images Using Nested U-Net Models	Applied Sciences (Switzerland), 13(23), 12771	2023	6
8.	Zhou X.	Tooth Type Enhanced Transformer for Children Caries Diagnosis on Dental Panoramic Radiographs	Diagnostics, 13(4), 689	2023	3
9.	Turosz N.	Oral Health Status and Treatment Needs Based on Artificial Intelligence (AI) Dental Panoramic Radiograph (DPR) Analysis: A Cross-Sectional Study	Journal of Clinical Medicine, 13(13), 3686	2024	2
10.	Asci E.	A Deep Learning Approach to Automatic Tooth Caries Segmentation in Panoramic Radiographs of Children in Primary Dentition, Mixed Dentition, and Permanent Dentition	Children, 11(6), 690	2024	2

**Table 2:** The selected articles from the second category.

Number	First Author	Title	Journal
1.	Endres M.G.	Development of a Deep Learning Algorithm for Periapical Disease Detection in Dental Radiographs	Diagnostics, 10(6), 430
2.	Zadrożny Ł.	Artificial Intelligence Application in Assessment of Panoramic Radiographs	Diagnostics, 12(1), 224
3.	Bayrakdar I.S.	A U-Net Approach to Apical Lesion Segmentation on Panoramic Radiographs	BioMed Research International, 2022, 7035367
4.	Song I.-S.	Deep learning-based apical lesion segmentation from panoramic radiographs	Imaging Science in Dentistry, 52(4), pp. 351–357
5.	Kazimierczak W.	Periapical Lesions in Panoramic Radiography and CBCT Imaging—Assessment of AI’s Diagnostic Accuracy	Journal of Clinical Medicine, 13(9), 2709
6.	Ba-Hattab R.	Detection of Periapical Lesions on Panoramic Radiographs Using Deep Learning	Applied Sciences (Switzerland), 13(3), 1516
7.	Güneç H.G.	Comparison of artificial intelligence vs. junior dentists’ diagnostic performance based on caries and periapical infection detection on panoramic images	Quantitative Imaging in Medicine and Surgery, 13(11), pp. 7494–7503
8.	İçöz D.	Evaluation of an Artificial Intelligence System for the Diagnosis of Apical Periodontitis on Digital Panoramic Images	Nigerian Journal of Clinical Practice, 26(8), pp. 1085–1090
9.	Turosz N.	Oral Health Status and Treatment Needs Based on Artificial Intelligence (AI) Dental Panoramic Radiograph (DPR) Analysis: A Cross-Sectional Study	Journal of Clinical Medicine, 13(13), 3686
10.	Herbst S.R.	Machine Learning to Predict Apical Lesions: A Cross-Sectional and Model Development Study	Journal of Clinical Medicine, 12(17), 5464

**Table 3:** The selected articles from the third category.

Number	First Author	Title	Journal	Year of Publication	Number of Citations
1.	Yang H.	Deep learning for automated detection of cyst and tumors of the jaw in panoramic radiographs	Journal of Clinical Medicine, 9(6), pp. 1–14, 1839	2020	112
2.	Okazaki S.	Analysis of the feasibility of using deep learning for multiclass classification of dental anomalies on panoramic radiographs	Dental Materials Journal, 41(6), pp. 889–895	2022	15
3.	Feher B.	Emulating Clinical Diagnostic Reasoning for Jaw Cysts with Machine Learning	Diagnostics, 12(8), 1968	2022	10
4.	Rašić M.	Detection and Segmentation of Radiolucent Lesions in the Lower Jaw on Panoramic Radiographs Using Deep Neural Networks	Medicina (Lithuania), 59(12), 2138	2023	7
5.	Tajima S.	Development of an automatic detection model using artificial intelligence for the detection of cyst-like radiolucent lesions of the jaws on panoramic radiographs with small training datasets	Journal of Oral and Maxillofacial Surgery, Medicine, and Pathology, 34(5), pp. 553–560	2022	5
6.	Ha E.-G.	Development of deep learning model and evaluation in real clinical practice of lingual mandibular bone depression (Stafne cyst) on panoramic radiographs	Dentomaxillofacial Radiology, 52(5), 20220413	2023	1
7.	Coşgun Baybars S.	Detection of Mucous Retention Cysts Using Deep Learning Methods on Panoramic Radiographs	Duzce Medical Journal, 26(3), pp. 203–208	2024	0
8.	Rašić M.	Utilizing Deep Learning for Diagnosing Radicular Cysts	Diagnostics, 14(13), 1443	2024	0

**Table 4:** Category 1—Dental Caries—the research objective, the number of panoramic radiographs, the distribution by gender and the location.

No.	Research Objective	Total Images	Gender Distribution	Research Location
1.	Assessment of AI reliability in panoramic image analysis.	30	16 women, 14 men	Medical University of Warsaw, Poland
2.	Development of an AI framework for diagnosing multiple dental diseases using panoramic images.	2278	Not specified	Zhejiang Chinese Medical University, China
3.	Use of a neural network to predict and detect radiation-related caries (RRC) based on PyRadiomics features.	420 teeth (15 patients)	13 men, 2 women	University of São Paulo, Brazil
4.	Development of AI for automatic tooth numbering and organisation of X-ray images into an FMS system.	1240	Not specified	San Jose State University, USA
5.	Development of an AI model for automatic segmentation of various structures in panoramic images to accelerate diagnostics.	8138	Not specified	Near East University, Cyprus
6.	Comparison of AI diagnostic efficiency with young dentists in detecting caries and periapical infections.	500	Not specified	Istanbul Medipol University, Turkey
7.	Use of U-Net models for detecting cavities in panoramic images.	1500	Not specified	Qassim University, Saudi Arabia
8.	Application of the Swin Transformer model for diagnosing caries in children using panoramic images.	6028	Not specified	Beijing Children’s Hospital, China
9.	Assessment of oral health in a population based on AI analysis of panoramic images.	980	Not specified	Kielce, Poland
10.	Evaluation of AI model effectiveness in segmenting caries in children’s panoramic images.	6075	Not specified	Ataturk University, Erzurum, Turkey

**Table 5:** Category 1—Dental Caries—the type of neural network, test quality, error rate, and the results.

No.	Type of Neural Network	Test Quality and Error	Research Findings
1.	Convolutional Neural Network (CNN)—Diagnocat	AI Sensitivity: 96.1% (missing teeth), 83.2% (fillings), 39.0% (periapical changes)	Diagnocat shows high specificity but low efficiency in assessing caries and periapical changes.
2.	BDU-Net, nnU-Net	AUC: 0.980 (impacted teeth), 0.772 (caries)	AI achieves high specificity but requires improvements in caries diagnosis.
3.	Artificial Neural Network (ANN)	AUC: 0.9869 (RRC detection), 0.9886 (RRC prediction)	AI achieved high sensitivity in detecting and predicting RRC.
4.	Deep learning framework	Accuracy: 95% (periapical images), 90% (bitewing images)	AI improves identification and diagnostic documentation of teeth.
5.	U-Net	Dice Similarity Coefficient (DSC): Teeth: 0.85, Caries: 0.88, Fillings: 0.87, Crowns: 0.93, Implants: 0.94, Root Fillings: 0.78, Residual Roots: 0.78	The AI model shows high effectiveness in segmenting various dental structures.
6.	CNN (Fast R-CNN, Faster R-CNN, SSD, YOLO)	AI Sensitivity for caries: 0.907, AI Sensitivity for periapical changes: 0.973, AI Specificity: 0.629–0.760	AI showed better effectiveness in detecting periapical changes than young dentists.
7.	Nested U-Net (U-Net, U-Net++, U-Net3+)	U-Net3+ achieved the best results with a test accuracy of 95%	The U-Net3+ model outperformed other U-Net versions in terms of caries detection accuracy.
8.	Swin Transformer	Accuracy: 85.57%, Precision: 88.32%, Sensitivity: 83.17%, F1-score: 85.67%, AUC: 92.23%	The Swin Transformer model outperformed classical CNN methods and showed better effectiveness in diagnosing caries in children.
9.	Deep learning model	AI achieved approximately 90% efficiency in detecting various pathologies	99% of participants had caries, 67% of patients underwent root canal treatment, 82% lost at least one tooth.
10.	U-Net (PyTorch)	Sensitivity: 0.8525 (deciduous teeth), 0.7377 (mixed), 0.8271 (permanent), Overall Accuracy: 0.8675	AI effectively detects caries in different types of children’s dentition.

**Table 6:** Category 1—Dental Caries—the risk of bias assessment using PROBAST.

Study	Participants	Predictors	Outcome	Analysis	Overall Risk of Bias
Zadrożny et al. (2022)	High risk	Unclear	Unclear	High risk	High
Zhu et al. (2023)	Low risk	Low risk	Low risk	Low risk	Low
Faria et al. (2021)	High risk	Unclear	Unclear	High risk	High
Kabir et al. (2022)	Low risk	Low risk	Low risk	Unclear	Low
Gardiyanoğlu et al. (2023)	Low risk	Low risk	Low risk	Low risk	Low
Güneç et al. (2023)	High risk	Low risk	Unclear	High risk	High
Alharbi et al. (2023)	Unclear	Low risk	Low risk	Low risk	Low
Zhou et al. (2023)	Low risk	Low risk	Low risk	Low risk	Low
Turosz et al. (2024)	Unclear	Low risk	Low risk	Unclear	Unclear
Asci et al. (2024)	Low risk	Low risk	Low risk	Low risk	Low

**Table 7:** Category 2—Periapical Lesions- the research objective, the number of panoramic radiographs, the distribution by gender, and the location.

No.	Research Objective	Number of Images	Gender Distribution	Research Location
1.	Development of a deep learning algorithm for detecting periapical lesions and comparison of its effectiveness with surgeons’ assessments.	2902 (training), 102 (test), 197 (validation)	No information	Harvard University, USA; Charité-Universitätsmedizin Berlin, Germany; Universitätsklinikum Hamburg, Germany
2.	Evaluation of AI effectiveness in panoramic radiograph analysis.	30	16 women, 14 men	Medical University of Warsaw, Poland
3.	Segmentation of periapical lesions based on AI.	470	No information	Eskisehir Osmangazi University, Turkey
4.	Use of neural networks for lesion segmentation.	1000	No information	Seoul National University, Republic of Korea
5.	Evaluation of AI effectiveness in diagnosing periapical lesions.	49 patients (1223 teeth)	No detailed data	Nicolaus Copernicus University, Toruń, Poland
6.	Development of an AI model for detecting periapical lesions and assessing its effectiveness.	713	No information	Qatar University, Doha, Qatar
7.	Comparison of AI and novice dentists in radiological diagnosis of caries and periapical infections.	500	No information	University of Health Sciences, Istanbul, Turkey
8.	Evaluation of AI effectiveness in diagnosing periapical inflammation based on panoramic radiography.	306	No information	Selcuk University, Konya, Turkey
9.	Assessment of oral health status and treatment needs using AI analysis of panoramic radiographs.	980	No information	Kielce, Poland
10.	Evaluation of factors related to the presence of periapical lesions and prediction of these lesions using ML.	1071 patients (27,532 teeth)	52.5% men, 44.8% women	Charité–Universitätsmedizin Berlin, Germany

**Table 8:** Category 2—Periapical Lesions—the type of neural network, test quality, error rate, and the results.

No.	Type of Neural Network	Test Quality and Error	Research Findings
1.	U-Net	AP: 0.60 (±0.04), F1-score: 0.58 (±0.04), PPV: 0.67 (±0.05), TPR: 0.51 (±0.05)	AI outperformed 14 out of 24 surgeons, but was not significantly better
2.	CNN	ICC: 0.619	AI had high specificity but lower accuracy for periapical lesions
3.	U-Net	F1-score: 0.88	AI detected 63 lesions on 47 radiographs
4.	CNN	F1-score: 0.828	AI detected 147 lesions in 180 cases
5.	Diagnocat AI software	F1-score CBCT: 84%, OPG: 32.73%	AI had high sensitivity (77.78%) in CBCT but low (33.33%) in OPG
6.	Two-step CNN model	AP detector: 74.95%, Classifier accuracy: 84%, Sensitivity: 72.2%, Specificity: 85.6%	AI demonstrated effectiveness in detecting lesions on panoramic radiographs
7.	AI model for radiological diagnostics	Sensitivity: 97.3%, Specificity: 62.9%, F1-score: 91.4%	AI outperformed novice dentists in detecting lesions
8.	Deep Learning (DL) model	Recall: 98%, Precision: 56%, F-measure: 71%	AI had high performance for the mandible but low for the maxilla
9.	AI Insights software (Carestream Health)	Image classification accuracy: 99%, Periapical lesion detection accuracy: 95%	AI enabled rapid screening of a large patient group
10.	Shallow ML models (logistic regression, k-NN, decision tree, random forest, SVM, boosting)	Best model effectiveness: Decision tree, F1-score 0.90	AI effectively predicted lesions based on radiographs

**Table 9:** Category 2—Periapical Lesions—the risk of bias assessment using PROBAST.

Study	Participants	Predictors	Outcome	Analysis	Overall Risk of Bias
Endres et al. (2020)	High risk	Low risk	Low risk	Unclear	High
Zadrożny et al. (2022)	High risk	Unclear	Unclear	High risk	High
Bayrakdar et al. (2022)	Low risk	Low risk	Low risk	Low risk	Low
Song et al. (2022)	Low risk	Low risk	Low risk	Low risk	Low
Kazimierczak et al. (2024)	High risk	Unclear	Low risk	High risk	High
Ba-Hattab et al. (2023)	Unclear	Low risk	Unclear	Low risk	Unclear
Güneç et al. (2023)	Unclear	Low risk	Low risk	Low risk	Low
İçöz et al. (2023)	Unclear	Low risk	Low risk	Low risk	Low
Turosz et al. (2024)	Low risk	Low risk	Low risk	Unclear	Low
Herbst et al. (2023)	Low risk	Low risk	Low risk	Low risk	Low

**Table 10:** Category 3—Cysts and Tumours—the research objective, the number of panoramic radiographs, the distribution by gender, and the location.

No.	Research Objective	Number of Images	Gender Distribution	Research Location
1.	Evaluation of the diagnostic effectiveness of the YOLO v2 neural network in detecting jaw cysts and tumours on panoramic radiographs.	1602	455 women (32%), 967 men (68%)	Yonsei University Dental Hospital, Republic of Korea
2.	Examination of the effectiveness of a neural network in classifying dental anomalies based on panoramic radiographs.	150	51 women, 99 men	Hiroshima University Hospital, Japan
3.	Reconstruction of the diagnostic process of maxillary cysts using artificial intelligence.	1239	No detailed data	Berlin (Germany), Nijmegen (The Netherlands), Cambridge (USA)
4.	Development of a deep learning model for automatic detection and segmentation of radiolucent lesions in the lower jaw.	226	No information	Clinical Hospital Dubrava and University of Zagreb, Croatia
5.	Development of an AI model for detecting cysts on panoramic radiographs using small training datasets.	7260	No information	Tokushima University Hospital in Japan
6.	Development of an AI model to distinguish pathological cysts from Stafne cysts.	1943	No information	Yonsei University Dental Hospital, Republic of Korea
7.	Application of deep learning methods for diagnosing and planning the treatment of mucous retention cysts in the maxillary sinuses.	161	No information	Fırat University, Faculty of Dentistry, Turkey
8.	Development of a deep learning algorithm for diagnosing radicular cysts based on panoramic radiographs.	238	No information	Clinical Hospital Dubrava, University of Zagreb, Croatia

**Table 11:** Category 3—Cysts and Tumours—the type of neural network, test quality, error rate, and the results.

No.	Type of Neural Network	Test Quality and Error	Research Findings
1	YOLO v2	Precision = 0.707, Sensitivity = 0.680	YOLO achieved the highest detection efficiency compared to maxillofacial surgeons and general practitioners, but the differences were not statistically significant.
2	AlexNet	Accuracy 70%, Precision 70.8%, Sensitivity 70%, F1-score 69.7%	The AlexNet network effectively classified cases of supernumerary teeth and odontoma.
3	Combination of object detection and image segmentation	Sensitivity 0.84 for odontogenic cysts, 0.56 for non-odontogenic; Specificity 0.59 for odontogenic cysts, 0.84 for non-odontogenic	The model was comparable to human specialists.
4	YOLO v8	Precision 95.2%, Sensitivity 94.4%, mAP@50 97.5%, mAP@50-95 68.7%	YOLO v8 effectively detected lesions in the lower jaw.
5	Deep Convolutional Neural Network (DCNN)	Accuracy 98.3%, Sensitivity 94.4%, Specificity 99.7%, Precision 99.0%, F-score 0.966	AI detected cysts with high accuracy.
6	EfficientDet	Accuracy 99.8%	AI effectively distinguished pathological cysts from Stafne cysts.
7	EfficientNet (B0–B7) + SVM	Accuracy: 0.878, Precision: 0.785, Sensitivity: 0.916, Specificity: 0.857, F1-score: 0.846	The EfficientNetB6 model showed the best efficiency in classifying mucous cysts.
8	YOLO v8 (with and without augmentation)	Without augmentation: Precision 85.8%, Sensitivity 66.7%, mAP@50 = 70.9%, mAP@50-95 = 60.2%; With augmentation: Precision 74%, Sensitivity 77.8%, mAP@50 = 89.6%, mAP@50-95 = 71.7%	The model effectively diagnosed radicular cysts, achieving high effectiveness thanks to the application of YOLO v8 and data augmentation.

**Table 12:** Category 3—Cysts and Tumours—the risk of bias assessment using PROBAST.

Study	Participants	Predictors	Outcome	Analysis	Overall Risk of Bias
Yang et al. (2020)	Low risk	Low risk	Low risk	Low risk	Low
Okazaki et al. (2022)	Unclear	Low risk	Low risk	Low risk	Low
Feher et al. (2022)	Low risk	Low risk	Low risk	Unclear	Low
Rašić et al. (2023)	Low risk	Low risk	Low risk	Low risk	Low
Tajima et al. (2022)	High risk	Low risk	Low risk	Low risk	Low
Ha et al. (2023)	Low risk	Low risk	Low risk	Low risk	Low
Cosgun Baybars et al. (2024)	Unclear	Low risk	Low risk	Low risk	Low
Rašić et al. (2024)	Low risk	Low risk	Low risk	Low risk	Low

**Table 13:** Presents a summary of the top-performing models in each category.

Category	AI Model	Accuracy/AUC	Sensitivity	Precision	Key Findings	Source Article
Dental Caries	PyRadiomics + ANN	AUC = 0.9886	High	High	Best results in detecting radiation-related caries	De Araujo Faria V. et al. (2021)
Dental Caries	U-Net3+ (Nested U-Net)	Accuracy = 95%	-	-	Top-performing among U-Net variants for caries detection	Alharbi S.S. et al. (2023)
Dental Caries	Swin Transformer	AUC = 0.9223	83.17%	88.32%	Outperformed classical CNNs in diagnosing children’s caries	Zhou X. et al. (2023)
Periapical Lesions	U-Net (Bayrakdar et al.)	F1-score = 0.88	92%	84%	Best segmentation of periapical lesions	Bayrakdar I.S. et al. (2022)
Periapical Lesions	Decision Tree	F1-score = 0.90	-	-	Best performance among classical ML models	Herbst S.R. et al. (2023)
Periapical Lesions	AI Insights (Carestream)	Accuracy = 99%	95%	-	Fast and precise detection, useful for screenings	Turosz N. et al. (2024)
Cysts and Tumours	EfficientDet	Accuracy = 99.8%	-	-	Highest performance in distinguishing pathological from Stafne cysts	Ha E.-G. et al. (2023)
Cysts and Tumours	YOLO v8	mAP@50 = 97.5%	94.4%	95.2%	Effective mandibular lesion detection with data augmentation	Rašić M. et al. (2023)
Cysts and Tumours	DCNN	Accuracy = 98.3%	94.4%	99.0%	Very effective in detecting cysts in large datasets	Tajima S. et al. (2022)

## Data Availability

No data from the authors’ studies were used in this work.

## Supplementary Materials

The following supporting information can be downloaded at: https://www.mdpi.com/article/10.3390/jcm14093262/s1, Prisma Flowchart.

## Abbreviations

The following abbreviations are used in this manuscript:

AI	Artificial Intelligence
ML	Machine Learning
U-Net	Convolutional neural network that was developed for image segmentation
CNN	Convolutional Neural Network
k-NN	k-Nearest Neighbors
SVM	Support Vector Machines
YOLO	You Only Look Once
AlexNet	convolutional neural network architecture developed for image classification tasks
DCNN	Diffusion-Convolutional Neural Networks
