# Supplementary figures and images for: Artificial Intelligence Methods in the Detection of Oral Diseases on Pantomographic Images—A Systematic Narrative Review
Source: J Clin Med. 2025 May 7;14(9):3262. doi: 10.3390/jcm14093262 (PMC12072333; doi:10.3390/jcm14093262)

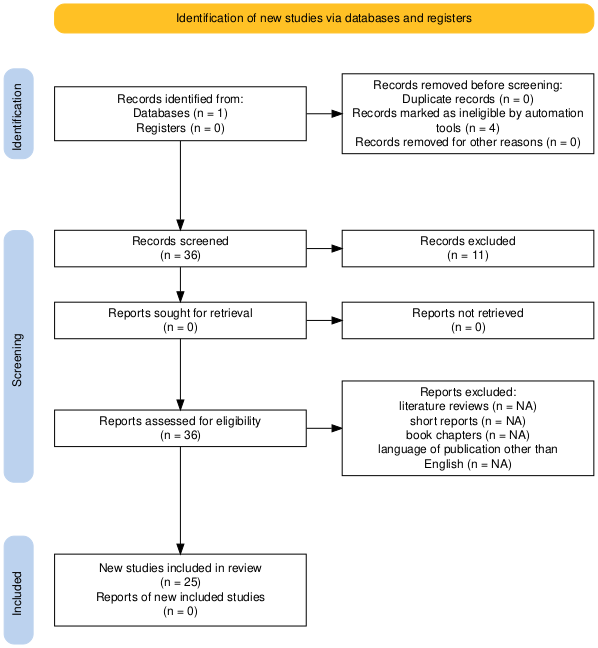

Supplement: Supplementary file 1 [file jcm-14-03262-s001.zip › jcm-3573708-supplementary.png]
